# Traditional Medications Mixed with Ethylene Glycol in a Nigerian Patient on Hemodialysis

**DOI:** 10.7759/cureus.6950

**Published:** 2020-02-11

**Authors:** Muhammad S Ajmal, Adebowale Awosika-Olumo, Rajeev Raghavan

**Affiliations:** 1 Nephrology, Baylor College of Medicine, Houston, USA; 2 Epidemiology, Walden University, Houston, USA

**Keywords:** traditional medicine, chronic kidney disease, herbal medicine

## Abstract

Unregulated traditional medications and their solvents are nephrotoxic. We present a case of a 49-year-old Nigerian male with a 10-year history of diabetes mellitus and hypertension who was ingesting a traditional, herbal medication as an aphrodisiac for erectile dysfunction. He had a rapid decline in kidney function over a period of one year and the patient commenced thrice weekly hemodialysis. He came to the USA for a second opinion. A full laboratory evaluation for immunologic and infectious causes of kidney failure was unremarkable. Kidneys were 12 cm bilaterally and a kidney biopsy revealed protracted tubular injury with isometric vacuolization and numerous calcium oxalate crystals. His serum oxalate level was elevated and there was no evidence of primary hyperoxaluria. It was suspected that the daily use of traditional, herbal supplements which often contain ethylene or diethylene glycol-based solvents may have led to a chronic oxalate toxicity that resulted in his kidney failure and above-mentioned pathological findings. Kidney damage was deemed irreversible and the patient returned to Nigeria. Worldwide, the increasing use of unregulated traditional, herbal supplements has the potential to cause epidemics of kidney disease in rural communities. A thorough medication history including the use of traditional and herbal supplements should be obtained in all patients with a rapid decline in kidney function, even in the presence of known risk factors for chronic kidney disease (CKD).

## Introduction

Chronic kidney disease (CKD) is a disease with a large global health burden and a high economic cost to health systems. The first systematic meta-analysis of CKD prevalence globally estimated CKD prevalence of 13.4% including stages 1-5 and 10.6% for stages 3-5 [1]. The prevalence of CKD in sub-Saharan Africa is believed to be high, but no estimate exists due to lack of systematic data collection [2]. The current incidence of 1.6%-12.4% reported by various studies is based on hospitalized patient data and thought to be low as many patients do not have access to the hospital [3]. These studies suggest that the peak incidence of CKD occurs in patients between 30-50 years of age [3].

Diabetes and hypertension are the most common causes of chronic kidney disease in North America and many developed countries [4]. Other causes of CKD vary by region of the world. For example, in Asia, glomerulonephritis including IgA nephropathy are common. In many developing countries, herbal and environmental toxins are believed to be a more likely cause of CKD [5]. Common causes of CKD in Nigerian adults are glomerulonephritis and hypertension, while common causes in children are glomerulonephritis and posterior urethral valves [3].

Traditional herbal medicines are naturally occurring, plant-derived substances with minimal or no industrial processing that have been used to treat illness within local or regional healing practices. These products are used widely in all countries; 80% of the African population use some form of traditional herbal medicine, and the worldwide annual market for these products approaches US$ 60 billion [6]. Herbal products are not classified as medication, hence, unregulated globally. There are varied reasons for the patronization of these products. The varied reasons include deep-rooted beliefs or superstition and perceived benefits. Herbal remedies are known to be mixtures of several herbal and non-herbal components. Nephrotoxicity has been associated with both the herbal and non-herbal components of the remedies. Such other components may be added deliberately or inadvertently due to poor handling techniques during preparation. Over three-quarters of the population in sub-Saharan Africa depends on traditional medicine using herbal remedies for primary health care [7-9]. Over 60% of the Chinese population use herbal therapy, while a significant part of the rural population in the Indian subcontinent relies on indigenous (Ayurvedic and Unani) medical systems that use herbs, ash, and heavy metals [8-10]. Various kidney diseases have been associated with traditional medicinal remedies [8]. These include acute kidney injury, tubular function defects, electrolyte imbalances, systemic hypertension, CKD, renal papillary necrosis, urolithiasis, and urothelial cancer [8,11-13]. Many edible herbs have been associated with CKD. For example, one identified toxic compound is aristolochic acid (AA). The AA-DNA adducts have been identified in the renal and urothelial tissues [8]. The following clinical features have been observed in aristolochic acid nephropathy (AAN) patients. These include progressive decline in glomerular filtration rate, evidence of proximal tubular dysfunction (glycosuria, increased excretion of low-molecular weight proteins), minimal albuminuria, absent edema and hypertension, severe anemia, metabolic acidosis, contracted kidneys and urothelial malignancies [8].

In sub-Saharan Africa, CKD is a very common non-communicable disease (NCD) with high morbidity and mortality. In most countries, people access both alternative medicine and biomedicine separately or consecutively for their healthcare needs. Little is known about the components of the alternative medicine dosages and side effects. Herein, we present the case of a Nigerian male who developed a rapid acute decline in kidney function following extensive patronization of herbal remedies in Nigeria.

## Case presentation

The patient is a 49-year-old Nigerian male with a 10-year history of diabetes mellitus and well-controlled hypertension. The patient and his personal physician traveled from Nigeria to Houston, TX for a second opinion. The patient’s hypertension and diabetes mellitus were well controlled. At the time of our evaluation, he was receiving thrice weekly hemodialysis for eight months through a cuffed dialysis catheter. He felt well and blood pressure was controlled, ultrafiltration amounts ranged from 2 to 4 liters per treatment.

For the last one year, he began daily ingestion of a traditional herbal remedy for erectile dysfunction. This traditional remedy was in liquid form and believed to be mixed with an alcohol-based solvent. He related that ingestion of such remedies was a customary practice in his village, outside Abuja, Nigeria. One year prior to presentation, the serum creatinine was 1.6 mg/dl with 0.8 gram/day proteinuria. Within one year, his serum creatinine increased to 8.9 mg/dl and the patient commenced thrice weekly hemodialysis at a local hospital in Abuja, Nigeria. He received dialysis thrice weekly for four hours per treatment and 2-4 liters ultrafiltration per treatment. His access was a cuffed catheter. His local physician was concerned, and the patient was sent to Houston, TX for a second opinion. Home medications included calcitriol, proton pump inhibitor (PPI), multivitamin, and a dipeptidyl peptidase 4 (DPP-4) inhibitor. His prior evaluation did include ultrasonography which demonstrated several non-obstructing kidney stones, but the patient denied any history of passing urinary stones.

**Figure 1:**
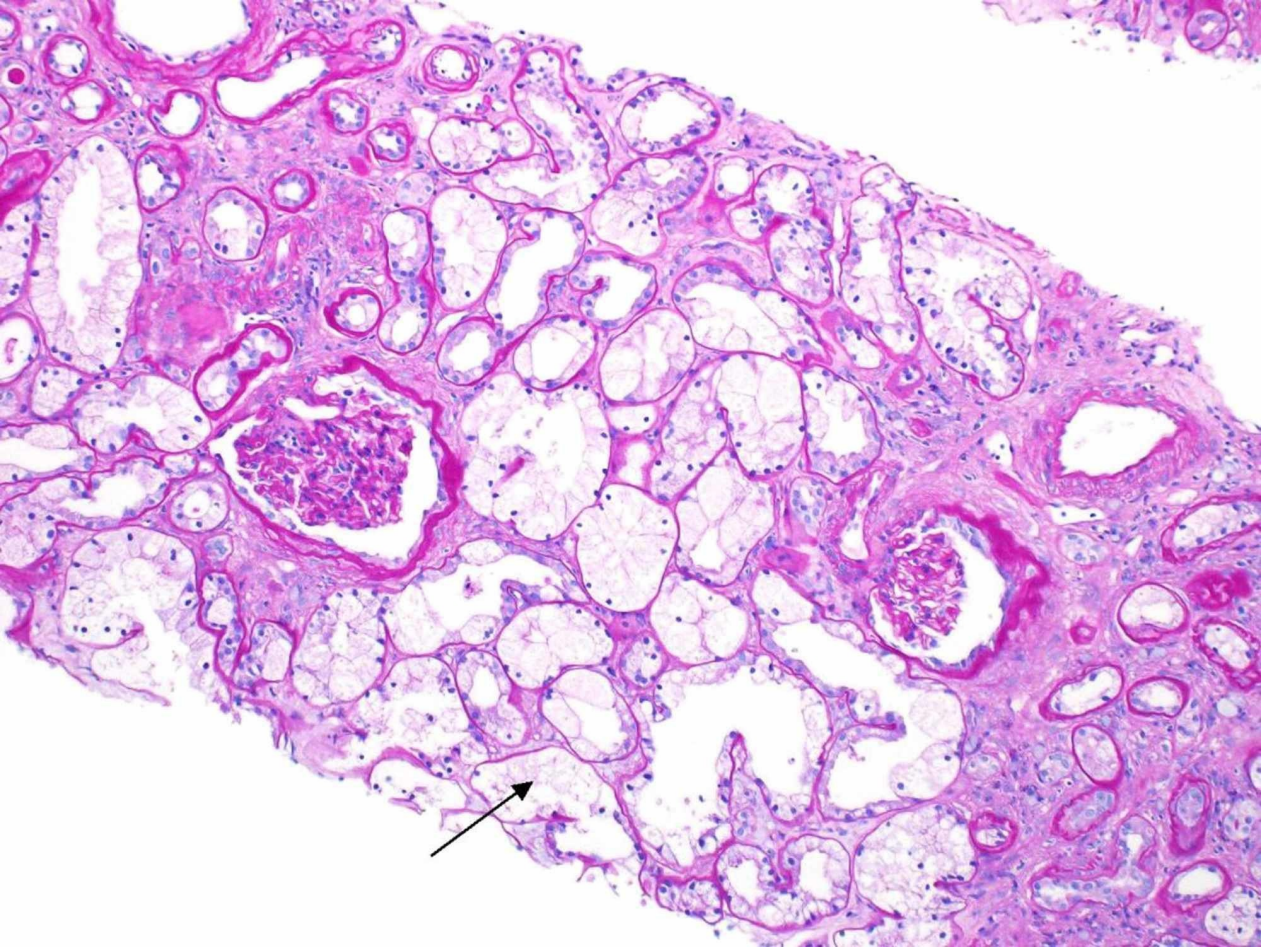
Light microscopy of kidney tissue showing protracted glomerular basement membrane, isometric vacuolization of the tubular cells, and interstitial fibrosis with tubular atrophy

**Figure 2:**
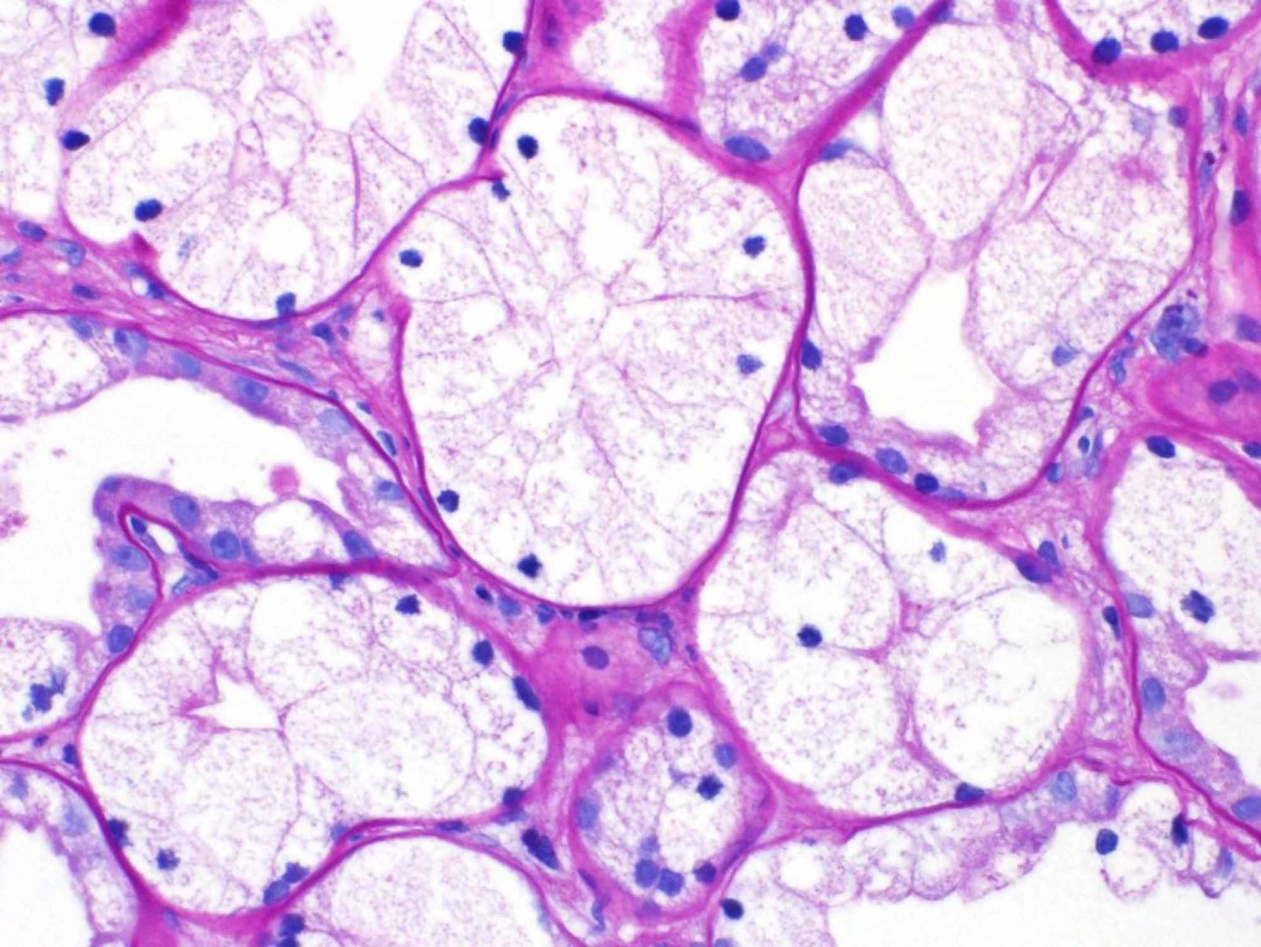
Light microscopy of kidney tissue showing protracted tubular injury with isometric vacuolization of the tubules

**Figure 3:**
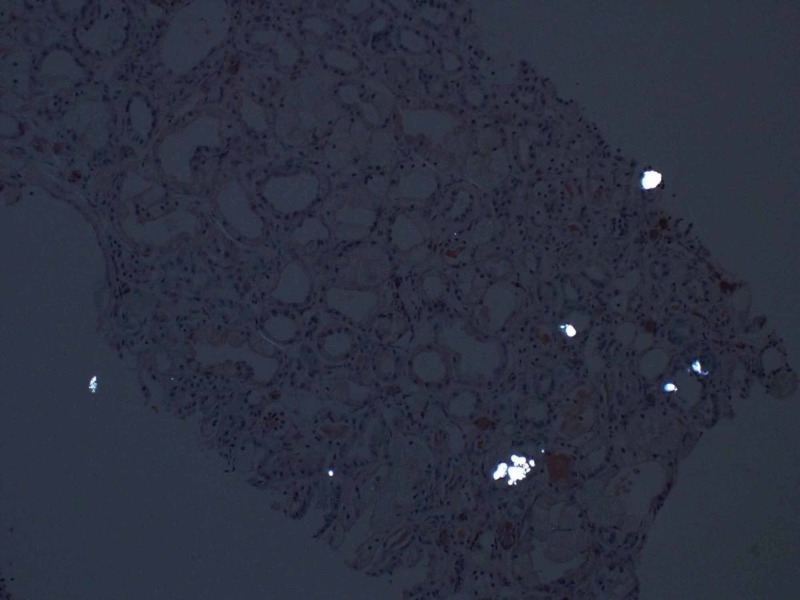
Calcium oxalate crystals visible on polarization

On physical exam, the patient has a blood pressure of 139/80 mmHg. Examination was largely unremarkable - there were no cardiac murmurs, lungs were clear, right internal jugular dialysis catheter site was clean, and there was absence of peripheral edema. A full 10-point review of the system was negative - pertinently, no ophthalmologic disease. Blood work including evaluation for autoimmune (vasculitis, lupus, Goodpasture's disease) and infectious causes (syphilis, human immunodeficiency virus (HIV), hepatitis) of kidney failure were negative. The patient was unable to provide a urine specimen. Ultrasonography showed kidney sizes 12.6 cm (R) and 11.1 cm (L) with normal echogenicity, no hydronephrosis, and only one non-obstructing kidney stone (7 mm) in the left kidney inferior pole was seen. There was an incidental finding of a 1.4 cm simple cyst in the right kidney. Subsequently, a kidney biopsy was carried out as part of the investigation. The biopsy revealed protracted tubular injury with isometric vacuolization and calcium oxalate crystals, 2/19 glomerulosclerosis, severe interstitial fibrosis and tubular atrophy, and severe arteriosclerosis (Figures 1-3).

The pattern of injury was not specific for a condition but associated with numerous agents including hypertonic solutions. The significant calcium oxalate crystals suggested various forms of hyperoxaluria. The degree of kidney disease was severe, and the patient likely would require life-long renal replacement therapy. Second, the lack of glomerular disease argued against diabetes as the primary insult. Third, the arteriosclerosis did suggest long standing hypertension as a significant contributor. Finally, the tubular damage and finding of oxalate crystals raised the possibility of hyperoxaluria. While his clinical history did not suggest primary hyperoxaluria (absence of other organ involvement), the suspected use of alcohol-based solvents (e.g. ethylene glycol) could have manifested with calcium-oxalate crystals. His serum oxalate level was elevated at 25 mcmol/L (range 1.8-3.2). There was no evidence of primary hyperoxaluria. His kidney damage was irreversible, and the patient returned to Nigeria.

## Discussion

Kidney disease is a major global public health threat. CKD and end-stage renal disease (ESRD) are prevalent in developing countries, most especially Nigeria [14]. Diabetes mellitus, hypertension and glomerulonephritis are reported common causes of CKD in Nigeria [14,15]. Worldwide, diabetes mellitus is the most common cause of chronic kidney disease, but in some regions of the world, other causes, such as herbal and environmental toxins, are more common [5]. Herbal medicines are but one component of complementary and alternative medicine (CAM), which includes acupuncture, chiropractic manipulation, meditation, reflexology, homeopathy, folk medicine and other cultural approaches [13]. In recent years, worldwide and including the US, herbal therapy has increased dramatically and has been associated with renal injury or various toxic insults [12]. In most countries, including the US, herbal remedies are not regulated as medicines. Alternative medicine and the use of herbal remedies are very popular among the poorer sections of society in the developing world. The source and composition of these medicines vary in different parts of the world, but herbs and other botanicals are central to these systems [7]. The herbal toxicity could be the direct impact of the herbs or may be secondary to the presence of undisclosed drugs or heavy metals, interaction with prescribed medications [12]. Various glycols including ethylene or diethylene glycol-based solvents are used as a preservative in preparation of traditional, herbal supplements [16].

An ethnomedical survey from North Tanzania identified five plant-based traditional medications used for kidney disease in the region, two of which have direct potential nephrotoxicity: Aloe vera and Cymbopogon citrullus [17]. In many instances, this nephrotoxicity is dose-dependent, and this underscores the additional importance of also understanding the mode by which people consume Traditional medicines because people with CKD may be particularly vulnerable to these effects. In the case of Aloe vera, which can cause acute tubular necrosis and acute interstitial nephritis in addition to chronic renal insufficiency, the nephrotoxicity is substantially higher with the larger doses ingested by boiling and drinking the plant [18]. Many edible plants have been associated with chronic kidney injury. These include Aristolochia spp., the toxic compound Aristolochic acid associated with chronic interstitial nephritis, renal tubular defects, and urothelial malignancies. Averrhoa carambola-- star fruit with toxic compound oxalic acid associated with nephrolithiasis and obstructive nephropathy. Furthermore, Vaccinium macrocarpon--cranberry with toxic compound oxalic acid, also associated with nephrolithiasis, and obstructive nephropathy [7].

The prognosis for CKD patients in Nigeria is abysmal. Only few patients had renal-replacement therapy (RRT). The prohibitive cost precludes many patients [2]. This underscores the need for preventive measures to reduce the impact of CKD in society. In most African countries, CKD and ESRD are very common in young adults in their economically productive years. The clinical pattern of disease is characterized by advanced CKD and acute kidney injuries secondary to preventable causes [18].

The current burden of CKD and ESRD in Africa and the unregulated patronization of alternative medicine and herbal remedies, along with poor diagnostic and renal therapies, demonstrates the need for global initiatives to prevent and manage kidney injury. The financial burden on the patients and the nation’s healthcare resources in the management of CKD and ESRD are the factors responsible for high morbidity and mortality of the disease in the developing countries. To control this current disturbing trend, there is need for an array of preventative actions which includes sensitization, continuous medical education, and strengthening of the health system to improve the management of patients with kidney disease; the implementation of a national program to prevent and control risk factors for kidney diseases cannot be understated.

## Conclusions

Worldwide, the increasing use of unregulated traditional herbal supplements has the potential to cause an epidemic of chronic kidney disease in rural communities. Various glycols including ethylene or diethylene glycol-based solvents are used as a preservative in preparation of herbal supplements. Frequent and excessive consumption of these supplements can lead to chronic oxalate toxicity that may result in kidney failure and pathological findings as described in our case. A thorough history and work-up should be pursued in all patients with a rapid decline in kidney function, even in the presence of known risk factors for chronic kidney disease. Increase awareness and possible regulations regarding the preparation of these supplements can help reduce the burden of CKD and end-stage kidney failure.
